# Charles Bonnet syndrome in patients referred for cataract surgery

**DOI:** 10.1007/s00417-026-07241-0

**Published:** 2026-04-22

**Authors:** Mads Assenholt Nielsen, Jakob Bjerager, Gülsenay Citirak, Yousif Subhi, Lars Morten Holm, Amardeep Singh

**Affiliations:** 1 Department of Ophthalmology, Rigshospitalet, Valdemar Hansens Vej 3, Glostrup, DK- 2600 Denmark; 2 Faculty of Health and Medical Sciences, University of Copenhagen, Copenhagen, Denmark

**Keywords:** Charles Bonnet Syndrome, Visual hallucinations, Cataract, Cataract Surgery

## Abstract

**Purpose:**

To investigate the prevalence and clinical characteristics of Charles Bonnet Syndrome (CBS) in patients referred for bilateral cataract surgery.

**Methods:**

This prospective, cross-sectional clinical trial included 391 patients attending cataract assessment at a single-center tertiary cataract clinic. After clinical examination, participants were screened for presence of visual hallucinations (VHs). Participants experiencing complex VHs were further questioned regarding characteristics of their hallucinatory experiences to properly determine if they were attributable to CBS. CBS was defined as complex visual hallucinations with retained insight, no medical history or medication known to cause hallucinations and not limited to hypnagogic or hypnopompic states (hallucinations happening shortly before falling asleep and/or shortly after waking up).

**Results:**

Nineteen (4.9%) patients experienced complex VHs. Of these, 11 (2.8%) were diagnosed with CBS while 8 (2.1%) had complex hypnagogic and/or hypnopompic VHs. Multivariable logistic regression analysis identified lower best-corrected visual acuity (BCVA) in the better seeing eye as a significant predictor of CBS. When excluding participants with a BCVA>0.3, prevalence of complex VHs rose to 10.0%. Three of the patients with CBS (27.3%) had ocular comorbidities: two had exudative age-related macular degeneration (AMD), and one had drusenoid AMD. In the overall cohort, twenty-four patients (6.1%) reported prior knowledge of CBS.

**Conclusion:**

CBS was observed in a clinically relevant minority of cataract patients requiring bilateral surgical intervention. Given the high prevalence of cataract among the elderly, this study highlights the importance of healthcare personnel being aware of CBS and other complex VHs and their potential consequences for cataract patients.

**Supplementary Information:**

The online version contains supplementary material available at https://doi.org/10.1007/s00417-026-07241-0.

## Abstract (fr)

**Key messages:**

*****What is known***:**

- Charles Bonnet Syndrome (CBS) is characterized by complex visual hallucinations in visually impaired patients and is mainly described in retinal and glaucomatous disease.

*****What is new***:**

- CBS was not uncommon among patients referred for bilateral cataract surgery.
- Most cases of CBS occurred without known or documented retinal pathology and was associated with lower visual acuity in thebetter-seeing eye.
- Accurate differentiation between CBS and other visual hallucination syndromes is crucial for valid prevalence estimates.

## Introduction

Charles Bonnet Syndrome (CBS) describes a phenomenon in which complex visual hallucinations (VHs) are induced by visual impairment [1–3]. Though definitions vary, CBS is usually described as a recurring experience of complex, vivid visual hallucinations due to loss of visual function in cognitively normal persons with preserved insight, not affected by neurological or psychiatric disturbances [2]. Despite retained insight into the unreal nature of their hallucinatory experiences, about a third of patients report subjective distress as a direct result of CBS [4, 5]. A recent systematic review and meta-analysis on worldwide presence of CBS estimates that one fifth of low vision patients or 47.2 million patients suffer from CBS [6].

Most commonly investigated in age-related macular degeneration (AMD) [7, 8] and glaucoma [9–11], CBS may present itself in many ocular pathologies, including diabetic retinopathy, retinal vein occlusion, retinitis pigmentosa, and Stargardt disease [2, 12–14]. Despite cataract being one of the most common causes of visual impairment, there is a lack of literature investigating CBS in cataract outside of case reports [15–17]. These reports describe patients with advanced cataracts in at least one eye, presenting with complex visual hallucinations and retained insight, including vivid scenes such as cults and devil worshippers, flying cars, and text on walls. In some cases, the hallucinations led to psychiatric evaluations before being attributed to CBS. Importantly, the symptoms were reported to resolve following cataract surgery in each of the cases.

Awareness of CBS has furthermore been shown to be low among both physicians [18] and patients [19]. With expanded knowledge of CBS, its clinical features and associated ocular pathologies, health care providers will be equipped to care for affected patients. The primary aim of this study is to investigate the prevalence and clinical characteristics of complex visual hallucinations in patients affected by bilateral cataract.

## Materials and methods

### Study design and population

This prospective, cross-sectional, single-center study was performed in adherence with the Declaration of Helsinki and all participants included provided written informed consent. According to Danish regulations, this observational study did not require approval by a regional ethics committee. Based on data from a similar clinical study in patients with AMD [20], we estimated that a minimum 300 patients were needed to detect a 5-fold difference in VH prevalence between groups of better seeing patients (estimated 2% prevalence) and worse seeing patients (estimated 10% prevalence) (80% statistical power; alpha 0,05).

Between September 7th, 2021 and July 4th, 2022, during a predefined study period in which financed personnel was available, 457 patients referred to the cataract clinic at the Department of Ophthalmology, Rigshospitalet, Glostrup, Denmark, were invited to take part in this study. During the inclusion period, a subset of participants was also enrolled in a separate clinical study on patient-reported outcomes of cataract surgery (*n* = 113) [21]. These patients were approached in parallel for participation in the present study. For practical reasons, patients excluded from this study due to study specific exclusion criteria (*n* = 8) were also excluded from the present study as clinical data such as visual acuity could not always be collected for them. In addition, the following exclusion criteria applied for the whole inclusion: Patients were excluded if they had hallucinations in sensory modalities other than vision, psychiatric or neurological conditions, substance abuse, or were taking medications known to trigger hallucinations. They were also excluded if unable to speak or comprehend Danish, or if presenting with acute conditions secondary to cataract, such as phacomorphic and phacolytic glaucoma. We did not set an upper limit of best-corrected visual acuity (BCVA), nor did we exclude patients with ocular comorbidities as we sought to investigate the prevalence of CBS in a patient population representative of a cataract clinic.

Due to the high volume of patients in the clinic, it was not possible to enroll all patients consecutively. Instead, the primary investigator enrolled as many patients as possible on designated days during the study period. Inclusion days were distributed across the study period to minimize seasonal or operational bias. Only patients eligible for bilateral cataract surgery were invited to join. As per the Danish national guidelines regarding cataract treatment [22], bilateral surgical intervention was indicated in patients presenting with either BCVA of ≤ 0.5 and/or significant subjective cataract-related complaints, including glare, difficulties reading, progressive lenticular myopia, monocular diplopia, as well as anisometropia.

**Fig. 1:**
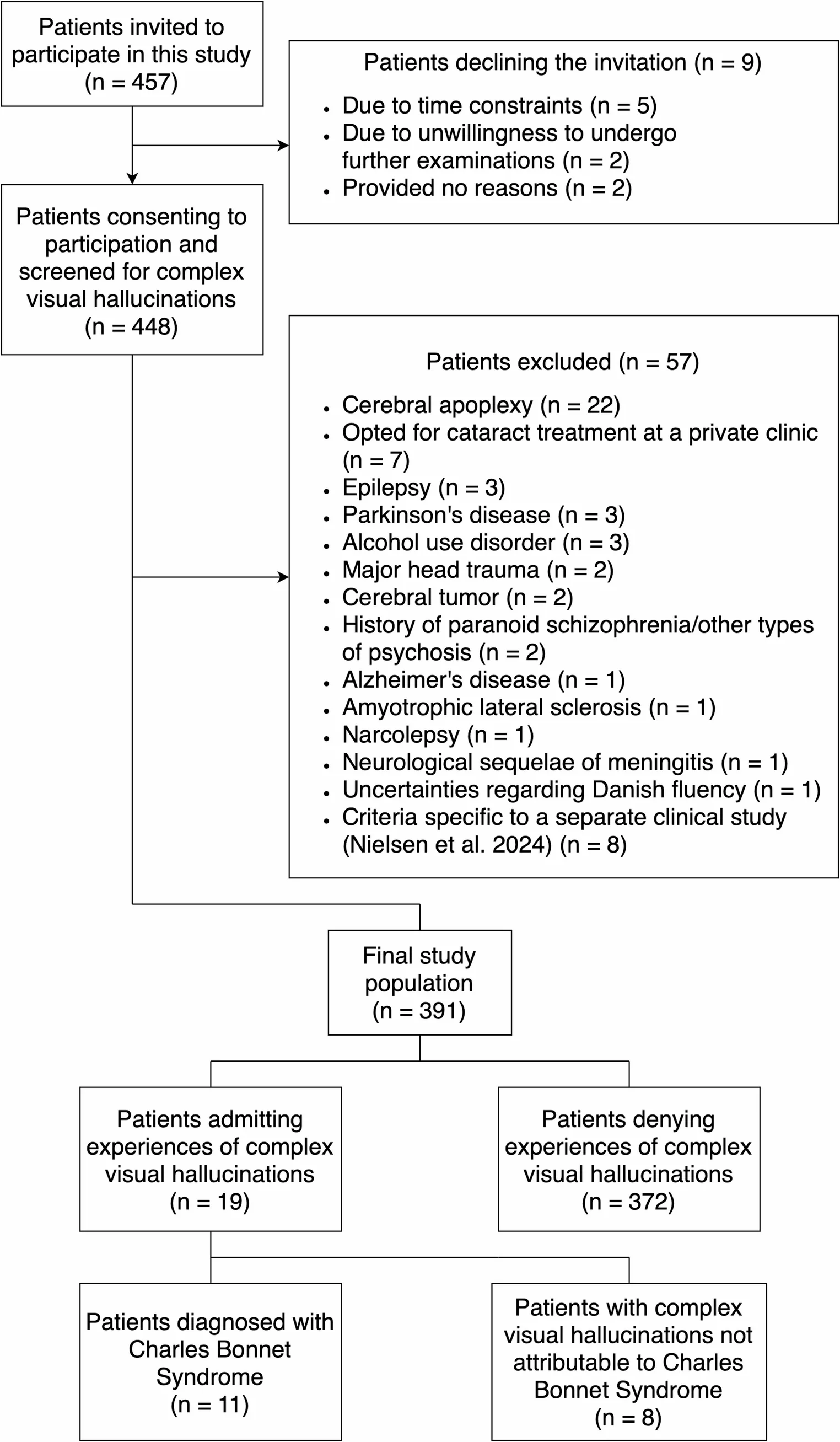
Flow chart illustrating the inclusion process

As a unified set of diagnostic criteria for CBS has yet to be established, and there is considerable heterogeneity in definitions of the condition across studies [23]. In line with criteria in some similar studies [7, 10, 20], we used the following criteria to differentiate CBS from other types of hallucinations:

i. Presence of complex VHs with no involvement of other sensory modalities (i.e. auditory or tactile hallucinations).
ii. VHs not exclusive to hypnagogic and/or hypnopompic states (hallucinations happening shortly before falling asleep and shortly after waking up, respectively.)
iii. Full insight to the unreal nature of the hallucinations.
iv. No history of substance abuse, medications or diagnoses known to cause hallucinations (e.g. schizophrenia, dementia; see Fig. 1 for full exclusion list).

Some other studies screen for mild cognitive impairment (MCI) [24, 25], which the present study did not. We also put additional emphasis on differentiating between sleep-related hallucinations and CBS.

Information on previous history of head trauma, neurological or psychiatric conditions, hearing impairment, and current medication was collected to rule out any other potential causes of hallucinations.

The presence of visual hallucinations was screened for by using the following question inspired by Holroyd [26] with slight modification and translated into Danish: *“Some people*,* with decreased eyesight like you*,* may experience visual hallucinations*,* meaning that they see things that are not really there. These may for example include people*,* animals*,* flowers or patterns. Have you ever experienced this?”*. All patients were asked by the same interviewer (M.A.N.) using the exact same phrasing to ensure consistency across all participants. All participants were also asked a screening question regarding prior knowledge of visual hallucinations caused by visual impairment: *“Did you*,* prior to this conversation*,* know that some people may experience visual hallucinations due to decreased eyesight?”*. Both questions had four response categories: *yes; no; maybe*,* I am not sure; I do not wish to answer*.

Participants who did not understand the questions immediately had the subject matter explained in greater detail. Patients admitting to having complex visual hallucinations were further questioned regarding the characteristics of their hallucinatory experiences. This assessment aimed to correctly classify patients with CBS and distinguish them from patients experiencing other types of hallucinations, i.e. sleep related hallucinations. See *Supplementary Material A* for the full questionnaire in its original Danish version. Patients only describing elementary visual hallucinations (such as shadows), entoptic phenomena (i.e. floaters) and/or illusions (mistaking one object for being another, e.g. mistaking a coat rack for being a person) were not counted as having complex VHs.

Patients diagnosed with CBS were provided with a detailed oral description of the condition, including reassurance that it is a recognized and benign phenomenon.

In all participants, refraction and BCVA (Snellen fraction afterwards converted to logMAR) was measured using KR-800 S Auto Kerato-Refractometer (Topcon, Tokyo, Japan); intraocular pressure (IOP) was measured using I-care (Vantaa, Finland); and Optical biometry was performed using IOL Master 700 (Carl Zeiss Meditec AG, Jena, Germany). In addition, a trial frame refraction was done in low vision patients (BCVA Snellen ≤ 0.5) and in cases where patient compliance with BCVA assessment by autorefractor had been judged insufficient by the operator. If deemed relevant, spectral domain optical coherence tomography (SD-OCT, Topcon, Tokyo, Japan) of the fundus was also performed. Slit-lamp examination including fundoscopy was performed to define the degree of lenticular opacity and to determine the status of the posterior segment. Use of proton pump inhibitors (PPI) was recorded, as this group of medication has been found to increase VHs [27, 28].

## Statistical

All analyses were conducted in RStudio (v2024.12.1; RStudio Team, 2022) using R version 4.4.1 (R Core Team, 2022). The Shapiro–Wilk test was used to assess normality. Continuous variables were summarized using median and interquartile range (IQR) and compared using the Mann–Whitney U test. Categorical data were analyzed using Fisher’s exact test.

Generalized linear models with a binomial family (logit link) were fitted to examine the presence of CBS as a function of covariates. First, a full multivariable model including logMAR BCVA of the better-seeing eye, logMAR BCVA of the worse-seeing eye, biological sex, age, and PPI use was constructed. Stepwise bidirectional model selection was then applied based on the lowest Akaike Information Criterion (AIC) to obtain a reduced model. Second, the presence of CBS was modeled using separate logistic regressions with BCVA of the better-seeing eye dichotomized at Snellen fractions of ≤ 0.1 (logMAR ≥ 1.00), ≤ 0.2 (logMAR ≥ 0.70), ≤ 0.3 (logMAR ≥ 0.52), ≤ 0.4 (logMAR ≥ 0.40), ≤ 0.5 (logMAR ≥ 0.30), and ≤ 0.6 (logMAR ≥ 0.22), each adjusted for sex.

Odds ratios (ORs) and 95% confidence intervals (CIs) were calculated using the Wald method. Statistical significance was defined as *p* < 0.05.

## Results

Of the 457 patients asked to participate, 9 declined the invitation. Of these, 5 declined due to time constraints, 2 due to unwillingness to undergo additional examinations and follow-ups, and 2 provided no reasons. Subsequently, 57 patients were excluded, mainly due to neurological and psychiatric conditions (Fig. 1).

In total, 391 individuals were included, of which 45 initially reported experiencing visual hallucinations. Upon further questioning about the nature of their hallucinations, 26 patients had experienced just simple hallucinations, entoptic phenomena (e.g. shadows and floaters) and/or illusions. Nineteen (4.9%) patients had experienced complex hallucinations. Of these, 11 patients (2.8% of the total study population of 391 patients) had experienced symptoms compatible with CBS, whereas 8 (2.1%) had experienced complex hallucinations only related to sleep (hypnagogic/hypnopompic hallucinations).

The median age was 76 years (IQR 8 years) for the total study population with 61% being females. There were no statistically significant differences regarding age, visual acuity of the better seeing eye, visual acuity of the worse seeing eye, distribution of sex, or of PPI use when comparing the group in which CBS was present to those that did not have CBS **(**Table 1**)**.

**Table 1:** Demographics

Variable	Total population	CBS absent	CBS present	CBS absent vs. present
n	391	380	11	-
Age, Median (IQR)	76 (8)	76 (8)	80 (10)	*p* = 0.254
Best seeing eye, LogMAR, Median (IQR)	0.222 (0.146)	0.222 (0.146)	0.222 (0.19)	*p* = 0.131
Worst seeing eye, LogMAR, Median (IQR)	0.398 (0.301)	0.398 (0.301)	0.301 (0.287)	*p* = 0.967
Sex, female	60.1%	60.8%	36.4%	*p* = 0.124
PPI use, yes	22.8%	22.6%	27.3%	*p* = 0.718

Abbreviations: CBS: Charles Bonnet Syndrome; IQR, interquartile range; PPI: proton pump inhibitor

Most cases of CBS were found at visual acuity of the best seeing eye of 0.6 Snellen (logMAR 0.22) (*n* = 5), followed by 0.5 and 0.3 (logMAR 0.52) (both *n* = 2). The case with the lowest visual acuity (0.02 Snellen; logMAR 1.70) had ocular comorbidity in the form of exudative AMD. Most patients diagnosed with CBS had no ocular comorbidity, though three cases included exudative AMD (*n* = 2), mild dry AMD with only drusen (*n* = 1). The clinical presentation of hallucinations among the 11 patients varied notably and included mostly human figures (*n* = 3) and environmental objects such as lamp posts, clocks and vehicles (*n* = 3). Some patients primarily described animals such as spiders and dogs (*n* = 2), while one patient described fantastical creatures such as dragons. Others described only complex colorful patterns (*n* = 2). Frequency varied from just once to daily occurrences with most patients experiencing hallucinations multiple times weekly. The duration was typically short (seconds to minutes), although few cases described hallucinations lasting multiple hours. Most patients experienced hallucinations in the afternoons and evenings **(**Table 2**)**.

**Table 2:** Characteristics of the 11 patients diagnosed with Charles Bonnet Syndrome

Patient	Content	Frequency	Time of day	Duration	Distress	Told anyone?	Duration of visual impairment	Onset
#1	Floating, illuminated lampposts when walking outside	Monthly	Early morning or evening	∼10 s	No	No	Six months	Six months ago
#2	A detailed, blue wire mesh fence covering specific walls, objects and people. Patient experiences the hallucinations during the interview	Daily, sometimes weekly	Afternoon or early evening	A few minutes	No	No	Several years	Five years ago
#3	Fails to mention any examples but insists on the presence of daily, complex hallucinations	Multiple times weekly	Morning	A few seconds	No	Yes; Family and general practitioner	Not specified	One year ago
#4	Wall clock with multiple protruding lights blinking in different colors	Once	Evening	∼10 s	No	No	Not specified	One day before the interview
#5	Colorful, small animals (spiders, butterflies) as well as flower bouquets	Multiple times weekly	At any time	Numerous seconds	No	No, afraid of telling others	Not specified	One and a half years ago
#6	A detailed red dog in the peripheral vision; different people (e.g. a woman wearing a dress)	Multiple times weekly, frequency is rising	Afternoon, evening	Indefinite: Until the patient looks directly at the visions	No	Yes; Family and nursing home staff	One year	Four months ago
#7	A complex pattern consisting of rhomboid shapes and rosettas with colored dots in them; covers walls, floors, objects and people	Weekly	Before noon	Several hours, sometimes the entire day	Yes; especially shortly after initial debut	Yes; Neighbor among other people	Not specified	Ten years ago
#8	Still-images of both living and dead family members	Multiple times weekly	Afternoon, evening	∼ 10 s	No	No	Two years ago	Six months ago
#9	Moving vehicles; food being cooked in their kitchen	Weekly	During the daytime	∼ 10 s	No	No	Between four and five years ago	One month ago
#10	Detailed roads appearing when driving in their car	Every time they are driving in the evening or at night	Evening, night	∼ 10 s	Yes; severe distress	Yes; Family and ophthalmologist	Ten years ago	Between six and eight years ago
#11	Fantastical creatures such as dragons and goblins	Multiple times per year	Evening	Minutes	No	Yes	Myopia since age 7	Many years ago

Two patients described distress, of which one reported severe distress due to the hallucinations. Just three patients had discussed their symptoms with either family members or health care professionals.

All patients diagnosed with Charles Bonnet Syndrome had preserved insight into the unreal nature of the hallucinations. None of the patients had experienced hallucinations of any other sensory modality (i.e. auditory).

Multivariable logistic regression analysis of presence of CBS as a function of all covariates found BCVA logMAR of best seeing eye to be the only statistically significant predictor of CBS (*p* = 0.03), with 1.0 increase in logMAR being associated with an odds ratio of 167 for CBS. Female sex was borderline significantly negatively associated with CBS (*p* = 0.057), OR 0.26. The best fitting model retained BCVA logMAR of best seeing eye, worst seeing eye and sex (*p* = 0.018, OR 255.75; *p* = 0.199, OR 0.07; and *p* = 0.05, OR 0.26 respectively) (Table 3).

**Table 3:** Logistic multivariable regression analyses of presence of CBS as a function of covariates

**Model/Predictor**	**Estimate**	**Std. Err.**	***p*-value**	**OR (95% CI)**	**AIC**
**Full model**					
Intercept	−6.47	3.55	0.068	< 0.01 [0.00–1.53]	100.43
Best seeing eye BCVA*	5.12	2.35	0.030	166.76 [2.98–9317.48]	
Worst seeing eye BCVA*	−2.43	2.11	0.248	0.09 [0.00–5.58]	
Sex (female)	−1.34	0.70	0.057	0.26 [0.07–1.00]	
Age	0.04	0.05	0.382	1.04 [0.94–1.15]	
PPI	−0.13	0.77	0.867	0.88 [0.20–3.77]	
**Best fit model**					
Intercept	−3.45	0.54	< 0.001	0.03 [0.01–0.10]	97.26
Best seeing eye BCVA*	5.54	2.34	0.018	255.75 [5.14–12720.58]	
Worst seeing eye BCVA*	−2.69	2.09	0.199	0.07 [0.00–4.14]	
Sex (female)	−1.36	0.70	0.051	0.26 [0.07–1.01]	

*for a 1.0 worsening of LogMAR. Abbreviations: AIC; Akaike Information Criterion; OR: Odds ratio; Std. Err.: Standard error

Multivariable logistic regression analyses of presence of CBS as a function of visual acuity thresholds and sex found the best model to be that including Snellen visual acuity of 0.3 or lower (logMAR 0.52 or higher). In this visual acuity group, the odds ratio of CBS was 5.87 (*p* = 0.017). Sex was still found to have borderline statistical influence in this model (*p* = 0.059). Snellen acuity of 0.1 or lower (logMAR 1.00 or higher) increased the odds ratio to 10.66 (*p* = 0.047), but it should be noted that only one CBS observation was found in this visual acuity group **(**Table 4**)**.

**Table 4:** Logistic multivariable regression analyses of presence of CBS as a function of visual acuity thresholds and sex

**Model/Predictor**	**Estimate**	**Std. Err.**	***p*-value**	**OR (95% CI)**	**AIC**
**Model 1**					101.04
Intercept	−3.1	0.39	< 0.001	0.05 [0.02–0.10]	
BCVA best seeing eye ≥ 1.00 logMAR (≤ 0.1 Snellen)	2.37	1.19	0.047	10.66 [1.04–109.50]	
Sex (female)	−1.12	0.66	0.087	0.33 [0.09–1.18]	
**Model 2**					102.07
Intercept	−3.1	0.39	< 0.001	0.04 [0.02–0.10]	
BCVA best seeing eye ≥ 0.70 logMAR (≤ 0.2 Snellen)	1.69	1.13	0.135	5.43 [0.59–49.86]	
Sex (female)	−1.08	0.65	0.094	0.34 [0.10–1.20]	
**Model 3**					99.09
Intercept	−3.23	0.41	< 0.001	0.04 [0.02–0.09]	
BCVA best seeing eye ≥ 0.52 logMAR (≤ 0.3 Snellen)	1.77	0.74	0.017	5.87 [1.38–25.01]	
Sex (female)	−1.26	0.67	0.059	0.28 [0.08–1.05]	
**Model 4**					103.23
Intercept	−3.16	0.42	< 0.001	0.04 [0.02–0.10]	
BCVA best seeing eye ≥ 0.40 logMAR (≤ 0.4 Snellen)	0.47	0.69	0.499	1.60 [0.41–6.22]	
Sex (female)	−1.02	0.64	0.11	0.36 [0.10–1.26]	
**Model 5**					103.48
Intercept	−3.17	0.47	< 0.001	0.04 [0.02–0.11]	
BCVA best seeing eye ≥ 0.30 logMAR (≤ 0.5 Snellen)	0.26	0.62	0.673	1.30 [0.39–4.35]	
Sex (female)	−1	0.64	0.114	0.37 [0.11–1.27]	
**Model 6**					99.29
Intercept	−4.44	1.03	< 0.001	0.01 [0.00–0.09]	
BCVA best seeing eye ≥ 0.22 logMAR (≤ 0.6 Snellen)	1.77	1.06	0.094	5.85 [0.74–46.38]	
Sex (female)	−1.05	0.64	0.1	0.35 [0.10–1.22]	

Abbreviations:AIC:Akaike Information Criterion; BCVA: Best-corrected visual acuity, CI: Confidence interval; Std. Err.: Standard error; OR: Odds ratio

## Discussion

This is the first study to investigate the prevalence and characteristics of Charles Bonnet Syndrome in the setting of cataract. We report that complex VHs are not a rare phenomenon among a large group of patients requiring bilateral cataract surgery, who are representative of the typical patient demographics in our clinic. VHs may occur when visual impairment arises due to cataract, either in isolation or in conjunction with other eye diseases. This study highlights the need for in-depth inquiry into the nature of hallucinations, since not all cases of complex VHs are a representation of CBS.

We saw a trend towards higher prevalence of VHs in association with lower visual acuity, with the prevalence of complex VHs rising to 10.0% when excluding participants with BCVA > 0.3. This association is in line with data from previous studies [29, 30]. Poor visual acuity does, however, not seem to be a prerequisite for developing of, as it has also been reported in patients with relatively well-preserved visual acuity [10, 24, 31]. Likewise, patients affected by complex visual hallucination in our study generally had relatively well preserved BCVA with just three patients affected by CBS having a BCVA of ≤0.3. While median BCVA did not differ significantly between the CBS and non-CBS groups, logistic regression analyses demonstrated a marked increase in the odds of CBS with lower BCVA, revealing a non-linear effect driven by poorer levels of visual acuity. The very large odds ratios observed in our models reflect the rarity of CBS events and the steep risk gradient across logMAR visual acuity levels and should be interpreted as indicative of strong association rather than precise effect size estimates.

Among the 391 included patients, prevalence of complex visual hallucinations was 19 (4.9%) with 11 (2.8%) being attributable to our definition of CBS. Since only four of the 19 patients experiencing complex VHs had ocular comorbidities, we conclude that the majority of complex VHs among our participants were most likely caused by cataract. Interestingly, eight of the 19 patients exclusively experienced complex hypnagogic and/or hypnopompic VHs.

In our multivariable logistic regression analysis, lower visual acuity in the best seeing eye was a significant predictor of CBS.

Just one participant experiencing complex VHs was previously familiar with CBS. Overall, prior knowledge of CBS was low among participants with just 24 out of 391 (6.1%) having heard of the condition. Previous studies have shown awareness of CBS to be low among both health care professionals [32] and patients [19, 33]. Reassurance and properly informing affected patients of the nature and course of the condition may reduce distress [2, 33]. Accordingly, it was prioritized that patients diagnosed with CBS were informed of the condition and reassured of its benign nature.

As only case reports have been made on CBS in cataract patients at this time, we can only compare our data with that of studies on CBS in other ocular diseases. Case reports do, however, describe complete remission of hallucinations in patients affected by CBS after surgical management [4, 15–17, 34]. This is of particular interest as CBS secondary to cataract may represent a potentially reversible form of the condition, in contrast with CBS associated with chronic ocular conditions such as AMD and glaucoma. As the present study was designed as a preoperative assessment, post-surgical changes in hallucinations were not systematically evaluated. In addition, the relatively small number of cases of CBS found in this study limited the possibility of performing meaningful analyses of post-surgical outcomes. Future longitudinal studies are needed to better characterize the effects of cataract surgery on CBS symptoms.

A recent systematic review and meta-analysis shows the overall prevalence of CBS among low vision patients ≥ 40 years to be 19.7% [6], highlighting the necessity of awareness towards the condition as well as proper care for affected patients. A more recent meta-analysis reported an overall CBS prevalence of 10.2% in a broad population of patients with eye disease, regardless of underlying diagnosis [35]. Both estimates are higher than the prevalence found in our study. This lower prevalence may partly be explained by our inclusion of patients in need of cataract surgery regardless of visual acuity in contrast to the meta-analysis, which focused solely on low-vision populations. The prevalence of CBS among our population (2.8%) is, however, in line with that of glaucoma patients in some previous clinical studies, with two studies respectively showing a prevalence of 3.1% and 2.2% [31, 36]. Another study [10] found a higher prevalence of 7.1% among patients with open-angle glaucoma.

In contrast, several clinical studies on CBS in patients suffering from AMD found a higher prevalence than that of our population [20, 24, 26, 37, 38], with a recent systematic review and meta-analysis showing an overall prevalence of CBS in patients with AMD of 15.8% [8].

While the exact physiology of CBS is not yet fully understood, the most widely accepted theory explained the condition is deafferentation. The theory suggests that reduced visual input increases excitability of the visual cortex, triggering spontaneous visual hallucinations [39–41]. In persons with normal vision, visual stimuli will inhibit this spontaneous activity. Functional magnetic resonance imaging (fMRI) studies have demonstrated this, showing a decreased cortical response to visual stimulus in patients visually hallucinating [42]. This suggests that when visual input is lacking, for example in AMD and glaucoma, endogenous visual hallucinations may be disinhibited. Recent studies have further examined the neurochemical basis of CBS. One such study investigated GABA as well as glutamate and glutamine concentrations in patients with CBS compared to controls with similar vision loss and found no significant differences in neurotransmitter concentrations, offering little support of the hyperexcitability hypothesis. The intensity of hallucinations was however linked to subtle changes in visual cortex activity, indicating that CBS may result from temporary changes in cortical excitability rather than fixed neurochemical imbalances [43].

Unlike AMD and glaucoma, cataracts do not damage the retina but may impair functional vision by reducing contrast sensitivity [44, 45] and image quality due to light scattering [44]. These changes may reduce the quality of functional visual input to a degree sufficient to induce CBS. Even without structural damage to the retina, the degraded visual input may lead to insufficient cortical stimulation, resulting in increased cortical excitability. Restoration of visual input following cataract surgery may then suppress this activity, which could explain the resolution of symptoms reported in some cases.

These mechanisms support the idea that CBS may occur in cataract patients when functional visual input is sufficiently impaired.

Some studies have reported female sex as a risk factor of CBS [24, 29]. Age has also been highlighted as a risk factor, with some studies finding a higher risk among the elderly [29] and others among younger patients [24]. Like some other studies [10, 20, 30], we did not find an association between CBS and age nor sex.

Some studies have shown oral PPIs to increase the risk of CBS in patients with AMD [27, 28]. We found no such association in our population, suggesting that this increased risk might not apply to CBS secondary to cataract. One study [28] put forth a hypothesis that access of PPIs to the outer retina due to breakout of the retinal barrier as well as blockage of feedback of horizontal cells to photoreceptors may explain the increased risk of hallucinatory activity in patients suffering with AMD. As cataract does not involve disruption of the blood-retinal barrier, this could explain why PPI usage does not seem to affect risk of CBS in our population.

CBS has been known to induce distress in about one third of affected patients [5, 20]. Just two of 11 patients affected by CBS in our population reported distress. This, however, does not necessarily challenge the data showing CBS to be distressful, as our population of affected patients is relatively small.

## Strengths and limitations

This study included a larger sample size than previous clinical studies on the prevalence of CBS in glaucoma and AMD [10, 34]. There was a high rate of study participation with just 9 of 457 patients (1.97%) declining the study invitation. This makes the study population fairly representative of the typical patient population at our clinic. Only one dedicated interviewer screened and interviewed all patients using the same screening questions and semi-structured interview format each time, ensuring consistency during inclusion. We used questions inspired by Holroyd [26] as other similar studies have done [20]. For all patients experiencing complex VHs, we asked about medication, prior head trauma as well as neurological and psychiatric diagnoses that could explain their hallucinatory experiences. All patient records were thoroughly examined to provide information on any such unmentioned diagnoses. With a recent systematic review [23] demonstrating a lack of unified diagnostic criteria for CBS, we carefully differentiated between simple hallucinations, illusions, hypnagogic and hypnopompic hallucinations, and CBS. As such, we are confident that 11 of our participants do, in fact, suffer from CBS.

Limitations include that we did not screen participants for MCI as some other studies have done [24, 25]. Instead, we relied on directly asking participants of any psychiatric diagnoses as well as post-hoc screening of patient records. Due to this, we may have overlooked undiagnosed dementia and other diseases that could possibly explain hallucinations or otherwise influence patient responses. We chose to only include patients with bilateral cataract as it has previously been proposed that bilateral disease increases likelihood of CBS [10, 37]. Recent reports have documented CBS following unilateral vision loss, suggesting that this may also be sufficient to trigger hallucinations [46]. Potential cases of complex VHs among patients with monocular cataract were missed, which could have affected the prevalence of VHs found. We cannot be certain that all participants answered our screening questions truthfully. In our study, nearly half of patients (nine out of 19) experiencing complex VHs had never told anyone about their experiences. This is a higher proportion than those found in some previous studies [20, 38].

We did not exclude patients based on relatively high visual acuity or presence of ocular comorbidities known to increase likelihood of CBS, such as AMD and glaucoma. As the primary aim of this study was to determine the prevalence of complex VHs in a clinical environment representative of a typical cataract clinic, we included patients with ocular comorbidities if cataract surgery was indicated. We did, however, not perform a systematic review of patient records for ocular comorbidities among those without VHs. Consequently, we cannot report the prevalence of CBS among patients verified to have cataract only. Future studies are thus needed to further investigate visual hallucinations among patients suffering exclusively from cataract. We did not perform contrast sensitivity testing on included patients. Reduced contrast sensitivity has been linked with an increased risk of CBS [47], while increased cataract severity has been linked with reduced contrast sensitivity [48].

In conclusion, our study found that complex visual hallucinations were observed in a clinically relevant minority of patients seen at a cataract clinic and may occur even when visual acuity is preserved. Some of these patients may suffer distress due to their hallucinatory experiences. Acknowledging and explaining the genesis of their visual hallucinations may help reassure these patients. Thus, health care professionals dealing with cataract patients should be aware of this condition.

## Supplementary Information

Below is the link to the electronic supplementary material.

Supplementary Material 1 (DOCX 107 KB)

## Data Availability

The datasets generated and analysed during the current study are not publicly available due to the sensitive nature of the data and patient confidentiality.

## Declarations

### Clinical trial number

Clinical trial number: not applicable.
