# Supplementary material for: Charles Bonnet syndrome in patients referred for cataract surgery
Source: Graefes Arch Clin Exp Ophthalmol. 2026 Apr 22;264(9):2783–93. doi: 10.1007/s00417-026-07241-0 (PMC13578024; doi:10.1007/s00417-026-07241-0)
Supplement: Supplementary file 1 [file 417_2026_7241_MOESM1_ESM.docx]

# **Supplementary material A**

## Patient questionnaire regarding Charles Bonnet Syndrome (CBS)

The original questionnaire was administered in Danish; the version below is provided as a direct English translation for reference.

**D. CBS screening and awareness**

Participant ID:	Date:

Investigator:

Some people with decreased eyesight experience visual hallucinations including things such as people, animals, flowers, or patterns that are not really there. This is a harmless condition caused by the brain generating images when it receives fewer signals from the eyes than normal.

**D1. Have you ever experienced visual hallucinations during the time you have had decreased vision?**

Yes (🡪 *follow-up questionnaire regarding CBS*)

No

Maybe, but I am not sure

I prefer not to answer

**D2. Before today, were you aware that some people with decreased eyesight may experience visual hallucinations?**

Yes

No

Maybe, but I am not sure

I prefer not to answer

**E. Follow-up Questionnaire regarding CBS (only in case of CBS)**

Participant ID:	Date:

Investigator:

**E1) Prior history of severe head trauma?**

□ Yes	□ No

**E2) Prior history of neurological or psychiatric disorder?**

□ Yes	□ No

If yes, which?

**E3) Hearing impairment?**

□ Yes	□ No

**E4) Current list of medication:**

**E5) What do the hallucinations consist of? (tick all boxes that apply)**

Trees, plants, flowers, or similar	□

Geometric figures	□

People	□

Animals	□

Other objects (e.g., buildings, vehicles, clothing, etc.)	□

Other (describe):	□

I don’t know / I prefer not to answer	□

**E6) How often do you see the hallucinations?**

Daily	□

Weekly	□

Monthly	□

Less often	□

I don’t know/I prefer not to answer	□

**E7) At what time of day do you usually see the hallucinations?**

Morning/before noon	□

Afternoon/evening	□

Anytime	□

At all times/Constantly	□

I don’t know/I prefer not to answer	□

**E8) Have you ever told anyone about the hallucinations?**

Yes – to whom:	□

No	□

I don’t know/I prefer not to answer	□

**E9) How long do the hallucinations usually last?**

Seconds	□

Minutes	□

Hours	□

I don’t know/I prefer not to answer	□

**E10) Do the hallucinations cause you any distress?**

No	□

Occasionally	□

To a large degree	□

I don’t know/I prefer not to answer	□

**E11) When did the hallucinations begin?**

Less than 12 months ago	□

More than 12 months ago	□

I don’t know/I prefer not to answer	□

**Anything else to add (e.g., further description of hallucinations or distress):**
